# Human serum activates the tegument of female schistosomes and supports recovery from Praziquantel

**DOI:** 10.1007/s00436-020-06968-x

**Published:** 2020-12-01

**Authors:** Franziska Winkelmann, Marcus Frank, Anne Rabes, Nicole Koslowski, Cindy Schulz, Miriam Bischofsberger, Emil C. Reisinger, Martina Sombetzki

**Affiliations:** 1 Division of Tropical Medicine and Infectious Diseases, Center of Internal Medicine II, University Medical Center Rostock, Rostock, Germany; 2 Medical Biology and Electron Microscopy Center, University Medical Center Rostock, Rostock, Germany; 3 Department Life, Light and Matter, University of Rostock, Rostock, Germany

**Keywords:** *Schistosoma mansoni*, Tegument integrity, Human serum, Ultrastructural analysis

## Abstract

Schistosomiasis is one of the most devastating parasitic disease in the world. *Schistosoma* spp. survive for decades within the vasculature of their human hosts. They have evolved a vast array of mechanisms to avoid the immune reaction of the host. Due to their sexual dimorphism, with the female worm lying within the gynecophoric canal of the male worm, it is the male that is exposed to the immediate environment and the soluble parts of the host’s immune response. To understand how the worms are so successful in fending off the immune attacks of the host, comparative analyses of both worm sexes in human serum (with or without Praziquantel) were performed using scanning electron microscopy, transmission electron microscopy, and immunohistochemistry. Further, gene expression analyses of tegument-specific genes were performed. Following the incubation in human serum, males and females out of pairs show morphological changes such as an altered structure of the pits below the surface and an increased number of pits per area. In addition, female schistosomes presented a marked tuft-like repulsion of their opsonized surface. The observed resistance of females to Praziquantel seemed to depend on active proteins in the human serum. Moreover, different expression profiles of tegument-specific genes indicate different functions of female_single and male_single teguments in response to human serum. Our results indicate that female schistosomes developed different evasion strategies toward the host’s immune system in comparison to males that might lead to more robustness and has to be taken into account for the development of new anti-schistosomal drugs.

**Supplementary Information:**

The online version contains supplementary material available at 10.1007/s00436-020-06968-x.

## Introduction

Schistosomiasis is considered the most important helminthic disease of humanity in terms of morbidity and mortality rates, affecting more than 200 million people mainly in the tropics and subtropics (Hotez et al. 2014). Estimates show that at least 229 million people required preventive treatment in 2018, out of which more than 97 million people were reported to have been treated (WHO 2018). Disease causing pathogens are parasitic flatworms of the genus *Schistosoma* spp. A characteristic of these parasites is their ability to survive for decades within the vasculature of their human hosts (Harris et al. 1984; Hornstein et al. 1990; Payet et al. 2006). Schistosomes exhibit a distinct sexual dimorphism between male and female worms. The female worm lies hidden in the gynecophoric canal of its male partner and is closely surrounded by it. Consequently, the tegument of the male worm is the first point of attack of the human immune system.

Matured schistosomes not only successfully fend off cellular and humoral immune, they use the host’s immune responses for their development and survival (Kusel et al. 2007; Maizels et al. 1993; McKerrow 1997; Pearce and MacDonald 2002; Pearce and Sher 1987). Schistosomes exhibit an astonishing variety of mechanisms that regulate their interactions with their host, including strategies of assimilation and attenuation of immune responses and the induction of immune tolerance that enable their long-term survival. Schistosomes can evade immune defense mechanisms in different ways. Well characterized is the antigen masking by the absorption of host serum proteins to the parasite surface (Loukas et al. 2001; Krautz-Peterson et al. 2017; Sepulveda et al. 2010). Second, molecular mimicry was considered as an important strategy to evade the host immune attack (Lehr et al. 2008; Salzet et al. 2000; Thompson 2001). Third, schistosomes are able to alter the host’s immune response, either directly through secretion of immune active proteins, or indirectly by deregulation of host effector cells and molecules (Han et al. 2009). It is known that they protect themselves from complement-dependent cytotoxicity by using inhibitory proteins like SCIP-1 (Angeles et al. 2020; Parizade et al. 1994). Other processes could also contribute to bypassing of the host immune system. For example, several enzymes involved in redox homeostasis, including glutathione-*S*-transferase, antioxidative thioredoxin peroxidases, and manganese superoxide dismutase, are located in the tegument (Liu et al. 2006). These enzymes are assumed to protect against environmental toxins, products of oxidative stress, and also innate immune attack through detoxification pathways (Kumagai et al. 2006; Loverde 1998; Mei and Loverde 1997; Sayed et al. 2006; Vermeire and Yoshino 2007; Williams et al. 2001).

The tegument of schistosomes plays a central role for the masking and therefore surviving within the host. It displays a continuous syncytium covering the entire outer surface of the worm. Numerous ridges and invaginations considerably enlarge the tegumental surface. A high concentration of different sensory papillae on the entire surface enables the worms to perceive a variety of stimuli from their environment. The tegument surface consists of two bilayers, an inner apical plasma membrane and an outer secreted membranocalyx. Below these surface membranes is the tegumental cytoplasmic layer. The tegument itself lacks many basic cellular components (e.g. ribosomes, nuclei, endoplasmic reticulum) and is connected via cytoplasmic bridges to thousands of individual cell bodies located under the muscle layers of the parasite. These tegumental cell bodies, called “cytons,” have nuclei and supply the tegument with proteins and secreted material to maintain its function (Wilson and Barnes 1974). Proteome analyses have shown that many schistosomal proteins such as transporters and enzymes are located within the inner membrane, whereas the host immunoglobulins and complement fragments are exclusively located in the outer membrane (Braschi and Wilson 2006). There is evidence that surface membrane turnover can be crucial for the immune evasion (Han et al. 2009). Previous work has focused on the immune evasion strategies of male worms (Han et al. 2009). Due to their hidden position, less is known about the evasion strategies of female worms.

It has been shown that the effectiveness of anti-schistosomal Praziquantel (PZQ) depends on the sex of schistosomes (Pica-Mattoccia and Cioli 2004). The mechanism of action of PZQ is not yet fully understood. However, early effects exerted by the drug can be summarized under three main headings: (1) calcium influx into the parasites, (2) muscle contraction, and (3) surface modifications (Cioli and Pica-Mattoccia 2003). It is most likely that calcium influx represents the key event, which in turn causes muscle contraction and tegument alterations. In vivo PZQ causes extensive structural changes to both male and female worms within 15 min of treatment. Nevertheless, variations in extent of drug-induced damage were observed in male and female worms. Apart of some tegumental vacuolization within the first 15 min, in female worms, the major structural changes are extensive vacuolization of the subtegumental tissue followed by varying degrees of structural damage of the subtegumental and gastrodermal musculature. In male worms, the initial effects are vacuolization of the dorsal tegument and loss of tegumental cytoplasm due to the pinching off of outer surface protrusions (Shaw and Erasmus 1983). The damaged tegument is utmost vulnerable to the host immune system.

The gender-specific differences in tegument alteration after PZQ treatment and the subsequent immune response of the host led to the assumption that female worms may have developed specific evasion strategies toward the host’s immune system. To test this hypothesis, we investigated whether human serum influences the male and female tegument differently. We used ultrastructural analysis and immunohistochemistry to visualize the changes in the tegument caused by human serum and tested the sex-specific motility of adult schistosomes in human serum in a time-dependent manner. Furthermore, we analyzed the gene transcription of tegument-specific proteins during incubation in human serum.

## Materials and methods

### Ethics statement

All animal experiments were performed in strict accordance with the regulations of the German Society for Laboratory Animal Science and with the European health guidelines issued by the Federation of Laboratory Animal Science Associations. The protocol was approved by the local committee on animal care and use (7221.3–2-022/17). All efforts were made to minimize animal suffering. Normal human serum (NHS) was obtained from a healthy donor, who has signed a declaration of consent. The research project was approved by the ethics committee of Rostock University Medical Center (A2018–0175).

### *Schistosoma mansoni* life cycle and worm isolation

*Schistosoma mansoni* (Belo Horizonte strain) was kept in a life cycle using *Biomphalaria glabrata* (*B. glabrata*) fresh water snails (Brazilian strain) as intermediate hosts and 6- to 8-week-old female NMRI mice as definitive hosts, as previously described (Sombetzki et al. 2015). To obtain either male or female cercariae for subsequent infection of mice, individual *B. glabrata* were exposed to single *S. mansoni* miracidia. Single sex cercariae were harvested 6 weeks later. The sex of the cercariae was determined by DNA amplification of sex-related chromosome segments using female-specific primers as previously described (Koslowski et al. 2017; Boissier et al. 2001). The mice were percutaneously infected with 300 *S. mansoni* cercariae, either male only, female only, or both. Adult worms were collected from infected mice at day 49 post infection by perfusion of the portal system with rinsing buffer [RPMI medium 1640 (Thermo Fisher Scientific, Germany), with 100 U/ml penicillin (Thermo Fisher Scientific, Germany), 100 μg/ml streptomycin (Thermo Fisher Scientific, Germany), and 1% heparin sodium salt (Sigma-Aldrich, Germany)]. Worms were washed three times with washing buffer (RPMI with 100 U/ml penicillin and 100 μg/ml streptomycin), and incubated in culture medium [RPMI with 100 U/ml penicillin, 100 μg/ml streptomycin, and 10% inactivated fetal bovine serum (Thermo Fisher Scientific, Germany)] at 37 °C in a humid atmosphere containing 5% CO_2_ until further use.

### Experimental groups

For the following experiments, isolated and separated female and male worms (female_pair, male_pair) were used as well as females and males out of a single-sex infection (female_single and male_single). These main comparison groups were selected to determine both gender-specific and mating independent effects. For further analyses, all experimental groups of ~60 adult worms each were exposed to undiluted normal human serum (NHS). As negative controls, we used the respective groups of worms incubated in heat inactivated serum (NHSi, heat-inactivated 30 min at 56 °C) as well as undiluted NHSi after overnight incubation in 10 μM Praziquantel (Sigma–Aldrich, Germany) (NHSi after PZQ) (Da'dara and Krautz-Peterson 2014). Positive controls for electron microscopy and motility assay were analyzed in undiluted NHS following overnight incubation in 10 μM Praziquantel (NHS after PZQ).

### Electron microscopy of the tegument of adult worms exposed to human serum

Female_single, male_single, female_pair, or male_pair were exposed to normal human serum (NHS) at 37 °C in a humid atmosphere containing 5% CO_2_ for 30 min followed by three times washing in PBS (pH 7.4; ThermoFisher Scientific, Germany). The respective control groups were analyzed in NHSi, NHSi after PZQ (negative controls), and NHS after PZQ (positive control) under the same conditions. After incubation within the respective medium and subsequent washing steps, the adult worms were stored in fixative solution containing 2% glutaraldehyde (EMS) and 1% paraformaldehyde in 0.1 M phosphate buffer pH 7.3 until further use. The worms were cut in two halves. One half was processed for transmission electron microscopy (TEM) and the other half for scanning electron microscopy (SEM). The medial posterior portion of five adult worms per group was analyzed at their cutting sites. From each group, the surface of three worms was imaged using SEM and one worm was analyzed via TEM. The specimen embedding for TEM involved a post fixation step using a buffered solution of 1% osmium tetroxide for 1 h followed by washing steps in distilled water and subsequent dehydration in an ascending series of acetone prior to the infiltration with epoxy resin (Epon 812, Serva, Germany) starting in a 1:1 mixture of acetone and resin overnight and with pure resin for 4 h. After transfer to rubber molds, the resin blocks were cured at 60 °C for 2 days. Both semithin sections (approximately 0.5 μm, stained with toluidine blue for light microscopy) and thin sections (50–70 nm, applied on copper grids with three slits for ultrastructural inspection) were cut on an ultramicrotome (Ultracut S, Reichert, Austria) with a diamond knife (Diatome, Switzerland). After treatment with uranyl acetate and lead citrate for contrasting, thin sections were examined in a Zeiss EM902 transmission electron microscope operated at 80 kV (Carl Zeiss AG, Germany). Digital images were acquired with a side-mounted 1x2k FT-CCD Camera (Proscan, Germany) using iTEM camera control and imaging software (Olympus Soft Imaging Solutions, Germany). For SEM preparation, worm tissues were dehydrated with a graded series of acetone for subsequent critical point drying using CO_2_ as an intermedium in an Emitech K850 critical point dryer (Emitech/Quorum Technologies Ltd., Laughton, UK). Specimens were flat mounted on SEM stubs with adhesive carbon tape (Plano, Germany) and sputter-coated with a gold layer (approximately 15–20 nm thickness) using a Bal-Tec SCD004 sputter coater (Balzers Union Ltd., Balzers, Liechtenstein). Specimen surfaces were analyzed with the field-emission SEM Zeiss Merlin VP compact (Carl Zeiss Microscopy, Germany) operated at 5 kV. Digital images with a size of 1024 × 768 pixels were recorded.

### Immunohistochemical staining of complement factor C3b bound to the tegument of adult worms exposed to human serum

Female_single, male_single, female_pair, or male_pair were exposed to normal human serum (NHS) at 37 °C in a humid atmosphere containing 5% CO_2_ for 30 min followed by triple washing with washing buffer. The negative control was incubated in heat-inactivated normal human serum (NHSi, 30 min at 56 °C) under the same conditions. After incubation, adult worms were incubated at room temperature for 30 min with 1 μg/ml mouse/anti human C3b antibody, clone H11 (Bio-Rad Laboratories, Germany). Adult worms were washed three times with washing buffer enriched with 0.2% bovine serum albumin (washing buffer + BSA) and incubated for 30 min at room temperature with 5 μg/ml a goat/anti-mouse IgG labeled with Alexa-Fluor 488 (Bio-Rad Laboratories, Germany). Following three times washing with washing buffer + BSA, the worms were transferred to PBS (pH 7.4; Thermo Fisher Scientific, Germany). Fluorescence signal was detected using a fluorescent microscope (Axio Scope.A1; Carl Zeiss Microscopy, Germany) equipped with an AxioCam MRc camera (Carl Zeiss Microscopy, Germany).

### Motility of adult worms exposed to human serum

For the assessment of the motility of adult worms, eight intact worms per group and time point were transferred into 96-well plates (Thermo Fisher Scientific, Germany), one single worm per well. Worms were incubated in 100 μl of NHS at 37 °C in a humid atmosphere containing 5% CO_2_. Controls were incubated in NHSi, NHSi after PZQ, and NHS after PZQ under the same conditions. The motility was monitored macroscopically after 0.5, 1, 24, and 72 h of incubation using a binocular microscope (Stemi 2000-C, Carl Zeiss Microscopy GmbH, Germany). Motility of worms was assessed using a viability scale of 0–3 described by Horiuchi et al. (2005): 3 = unaffected body movement; 1.5 = partial body movement; 0 = no worm movement observable for more than 2 min.

### Gene expression analysis of tegument-specific genes after exposition of adult worms to human serum

Adult worms were exposed to NHS or to NHSi (control) for 0.5, 1, 24, and 72 h. Five worms were analyzed for each time point. Worms were frozen (− 80 °C) in lysis buffer (RNeasy Micro Kit, Qiagen, Germany) until further usage. Five worms were pooled for RNA isolation (one biological replicate). RNA was isolated (RNeasy Micro Kit) and quantity of RNA was measured on a Colibri Microvolume Spectrometer (Titertek-Berthold, Germany) and 500 ng of total RNA was used to be reversely transcribed into cDNA using High-Capacity cDNA Reverse Transcriptase Kit (ThermoFisher, Germany) according to the manufacturer’s instructions. All primers and probes were purchased from Eurofins Genomics, Germany (Table S1). Gene candidates have been selected with regard to their suspected involvement in tegument integrity, renewal, or repair processes of the tegument or immune evasion strategies of adult schistosomes. As an endogenous control, we used the housekeeping *S. mansoni* alpha tubulin gene for relative quantification. Each qRT-PCR reaction was performed using 2 μl of the cDNA, in a final volume of 10 μl. All samples were run in triplicate. QPCR was performed using the QuantStudio 3 (Thermo Fisher Scientific, Germany) under the following reaction conditions: 50 °C for 2 min followed by 95 °C for 10 min, 45 cycles at 95 °C for 15 s, and at 59 °C for 1 min. The ΔΔCt method was employed for relative quantification (Livak and Schmittgen 2001). For graphical representation, ΔΔCt values in NHS were normalized to the endogenous control and presented as normalized, expression values of NHSi controls.

### Statistics

Statistical analysis was performed using GraphPad Prism 5.0 (GraphPad Software, La Jolla, CA, USA). Values are expressed as mean + SE_MEAN_. The samples were compared using the Kruskal-Wallis test followed by a Dunn’s correction. For all statistical analyses, *p* values <0.05 were considered significant (# *p*_Kruskal-Wallis_ < 0.05).

## Results

### Ultrastructural analysis of human serum-induced surface modifications in adult male and female *S. mansoni*

In order to investigate phenotypical changes due to the incubation in human serum, the tegument morphology of the adult worms was evaluated by scanning electron microscopy (SEM) and transmission electron microscopy (TEM). The medial posterior portion of the tegument was observed for each group of adult worms.

**Fig. 1:**
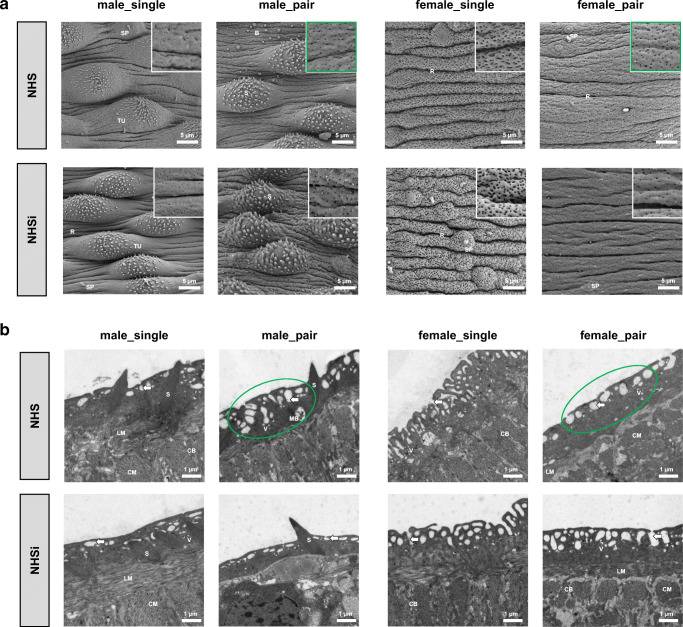
Ultrastructural analysis of surface modifications in adult male and female *S. mansoni* after incubation in human serum. **a** Scanning electron microscopy (SEM; 2500x) with details of tegument structure (10,000×) and **b** transmission electron microscopy (TEM; 7100×) of the medial posterior portion of adult male (male_single, male_pair) and female (female_single, female_pair) *S. mansoni* after 0.5-h incubation in NHS and NHSi. Presence of tegumental ridges (R), tubercles (TU), spines (S), sensory papillae (SP), blebs (B), circular musculature (CM), longitudinal musculature (LM), cytoplasmic bridge (CB), membranous body (MB), vesicles (V), and pits of the outer surface (arrows) are indicated. Representative pictures out of five adult worms per group. Conspicuous areas as a result of NHS treatment encircled in green

**Fig. 2:**
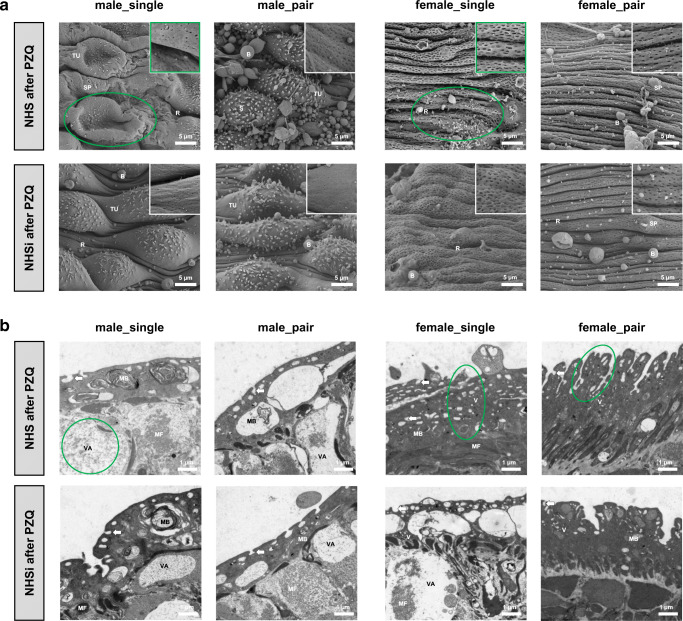
Ultrastructural analysis of surface modifications in adult male and female *S. mansoni* after Praziquantel treatment. Scanning electron microscopy (SEM; 2500×) with details of tegument structure (10,000×) and transmission electron microscopy (TEM; 7100×) of the medial posterior portion of adult male (male_single, male_pair) and female (female_single, female_pair) *S. mansoni* after 0.5-h incubation in **a** NHS after PZQ and **b** NHSi after PZQ. Presence of tegumental ridges (R), tubercles (TU), spines (S), sensory papillae (SP), blebs (B), muscle fibers (MF), membranous body (MB), vacuoles (VA), vesicles (V), and pits of the outer surface (arrows) are indicated. Representative pictures out of five adult worms per group. Conspicuous areas as a result of NHS treatment encircled in green

Tegumental ridges, some sensory papillae, and numerous pits characterize the outer surface of the adult worms in NHS (Fig. 1a). Both groups, worms out of pairing, male_pair and female_pair, showed an increased number of pits per area in NHS compared to NHSi. Below the surface, changes due to the incubation in NHS could be detected in these groups: male_pair showed deeper pits, while female_pair displayed more shallow pits (Fig. 1b). NHS after PZQ causes considerably less pits per area at the surface of male_single and female_single compared to NHSi after PZQ (Fig. 2). Furthermore, the tubercles of male_single appeared to be collapsed in NHS after PZQ. The tegumental ridges of female_single in NHS after PZQ have a regular and thin appearance with undefined irregularities, while in NHSi after PZQ the surface seemed to be inflated but intact. Female_pair showed thinner tegumental ridges in NHS after PZQ compared to NHSi after PZQ (Fig. 2a). When looking at the structures below by TEM (Fig. 2b), in male worms the degeneration of the tegumental and subtegumental layers and muscle shrinkage with widespread vacuolization as well as large membrane bodies were visible. The vacuolization is most pronounced in male_single in NHS after PZQ and, surprisingly, also found in female_single after incubation in NHSi after PZQ. In contrast, the tegument of female_single in NHS after PZQ is profuse with deep reaching pits and apparently intact subtegumentary region. TEM imaging for female_pair in NHS after PZQ revealed the thinner tegumental ridges compared to NHSi after PZQ.

In conclusion, the sex-specific morphological differences observed in this study seem to depend more on mating status than on active serum proteins (Fig. 1a). Female adult worms showed a pronounced enlargement of their surface structure compared to the males. Female_single displayed the highest enlargement with significantly more slit-shaped pits and deeper tegumental ridges, while female_pair worms displayed a smoother surface with flatter ridges and less rounded pits. In contrast to the females, the surfaces of male worms displayed well-developed tubercules with spines.

### Female schistosomes show a marked tuft-like repulsion of their opsonized surface after incubation in human serum in vitro

**Fig. 3:**
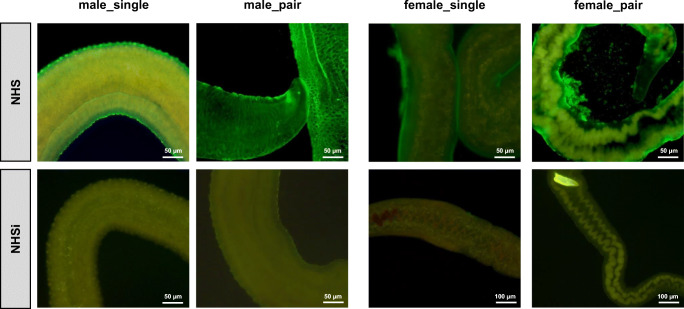
Female schistosomes show a marked tuft-like repulsion of their opsonized surface after incubation in human serum in vitro. Fluorescence microscopy (100×: females in NHSi, 200×:) of adult male (male_single, male_pair) and female (female_single, female_pair) *S. mansoni* after 0.5-h incubation in NHS or NHSi with following immunohistochemical staining of human complement factor C3b

For visualization of reorganization processes of the tegument, we performed immunohistochemistry for complement factor C3b at the surface of adult schistosomes after incubation in human serum. Female schistosomes presented a marked tuft-like repulsion of their opsonized surface compared to male and the NHSi control. It appears that part of the surface, made visible by fluorescence marked C3b, is peeling or repelled. The repulsion is most pronounced in female_pair worms and absent in the male groups (Fig. 3).

### Human serum has a regenerative effect on adult male and female *S. mansoni* following Praziquantel challenge in vitro

We analyzed the motility of adult worms as marker for their viability in the respective medium. As demonstrated in Table 1, analysis of the motility revealed no differences for adult worms treated with normal human serum (NHS). Throughout the measurement (0.5, 1, 24, and 72 h), NHS incubated worms showed peristaltic movements and characteristic waves through the body, suckers in constant movements, and temporarily adherence to the plate bottom (score = 3). In contrast, adult worms in NHS after PZQ displayed a restricted motility at the earlier time points. Slow movements or intestinal peristalsis were seen by all the female groups after 0.5 h in NHS (score = 1.5), although they appeared contracted and curled. In contrast, during the first hour of observation, both the male groups, male_single and male_pair, showed no body movement (score = 0), appear heavily contracted shaped as half-moons. Fifty percent of female_single displayed a score of 1.5 after 1 h in NHS, whereas the male groups reached this score only after 24 h. After 24 h, female worms showed a normalized motility as well as normal body shape and length. Contrary to this, the male groups displayed at this time point a slightly movement of some body parts (score = 1.5), such as the oral suckers and/or the posterior extremity. Interestingly, adult worms in NHSi after PZQ did not show any movement within the first 24 h. After 24 h 50% of female_single and 62.5% of female_pair displayed a score of 3.0, whereas the male groups did not reach this score in NHSi after PZQ. Male_pair showed no motility at all in NHSi after PZQ, while 50% of male_single showed restricted to completely inhibited motility over the course of time. In females, no motility was observed after 72 h in 50% of female_single and for 100% of female_pair. In summary, contrary to our expectations, NHS seemed to have a positive effect on the motility of adult schistosomes after pre-incubation with praziquantel. In addition, female schistosomes regenerate faster in NHS and also in NHSi after pre-incubation in Praziquantel compared to male worms.

**Table 1:** Motility in human serum. Motility scores for adult worms in vitro treated with normal human serum (NHS), inactivated normal human serum (NHSi), NHSi after overnight-preincubation with Praziquantel (NHSi after PZQ), and NHS after overnight-preincubation with Praziquantel (NHS after PZQ) at different time points (*n* = 8)

		Percent of worms (%) in motility score after incubation											
Groups	*t*_incubation_	0.5 h			1 h			24 h			72 h		
	Score	3	1.5	0	3	1.5	0	3	1.5	0	3	1.5	0
NHS	Male_single	100			100			100			100		
	Male_pair	100			100			100			100		
	Female_single	100			100			100			100		
	Female_pair	100			100			100			100		
NHSi	Male_single	100			100			100			100		
	Male_pair	100			100			100			100		
	Female_single	100			100			100			100		
	Female_pair	100			100			100			100		
NHS after PZQ	Male_single			100			100		12.5	87.5		62.5	37.5
	Male_pair			100			100		12.5	87.5		25	75
	Female_single		12.5	87.5		50	50	100			100		
	Female_pair		25	75			100	50	50		100		
NHSi after PZQ	Male_single			100			100		50	50		37.5	62.5
	Male_pair			100			100			100			100
	Female_single			100			100	50	25	25	50		50
	Female_pair			100			100	62.5	25	12.5			100

3, movement of the whole body; 1.5, movement of parts of the body or slowed movement; 0, no movement

### Expression profiles of tegument-associated genes indicate different functions of female_single and male_single teguments in response to human serum

**Fig. 4:**
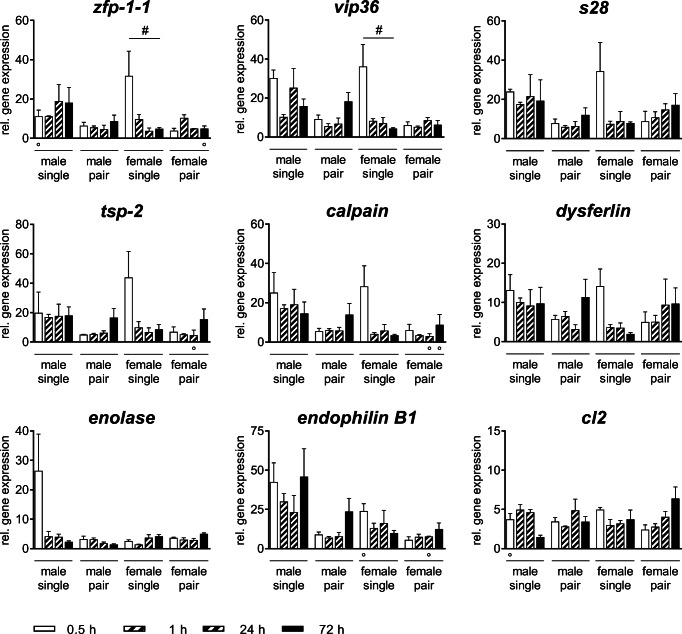
Human serum differentially impacts gene expression profiles of male and female *S. mansoni*. Relative gene expression of Calpain, SmCL2-like peptidase (CL2), Dysferlin, EndophilinB1, Enolase, Family S28 unassigned peptidase (S28), Tetraspanin-2 (TSP-2), Vesicular integral-membrane protein 36 (mannose-binding lectin 2)-related (Vip36), and Zinc finger protein-1-1 (ZFP-1-1) treated with normal human serum (NHS) at different time points was determined by real-time PCR. Data are presented as mean + SE_MEAN_; *n* = 3 (○ 2) each group; # *p*_Kruskal-Wallis_ < 0.05

Expression levels of genes associated with the tegument were analyzed in adult male and female schistosomes incubated with NHS (Fig. 4) at different time points. The expression of all tested genes in all experimental groups (male_single, male_pair, female_single, female_pair) is upregulated in the presence of NHS. In female_single, significant differences were detected for the expression of two genes over time: *zinc finger protein*-*1*-*1* (*zfp*-*1*-*1*) and *vesicular integral*-*membrane protein 36* (*vip36*) were initially upregulated by incubation in NHS (0.5 h) followed by a rapid decrease of the gene expression levels. A comparable trend was observed for *family S28 unassigned peptidase* (*s28*), *tetraspanin-2* (*tsp-2*), *calpain* and *dysferlin*, with strongest expression levels in female_single worms. In contrast, the expression of *enolase* was only upregulated in male_single after 0.5 h in NHS. Interestingly, while the adult worms out of a single-sex infection, female_single and male_single, show changes in the gene expression in NHS over time, but no significant differences were found in adult worms out of pairs, female_pair and male_pair. The expression of *endophilin B1* and *smcl2*-*like peptidase* (*cl2*) did not show any significant differences within the experimental groups in NHS over time.

## Discussion

This study was conducted to characterize the effect of human serum (NHS) on the tegument turnover/replacement of female schistosomes compared to male schistosomes. Immunohistochemical staining for complement factor C3b revealed a binding of C3b to female and male teguments. However, a marked shedding of the tegument was exclusively observed in female worms, most pronounced in the female_pair group (Fig. 3). Herein, we demonstrated that male_pair and female_pair display morphological changes at their surfaces following incubation in NHS shown by an increased number of pits per area of tegument and below. Furthermore, we could show that female worms are significantly more resistant to incubation with PZQ than male worms, regardless of whether they originate from a single-sex or natural infection. Interestingly, NHS seems to promote regeneration of the worms after a PZQ challenge, especially in regard to the female worms. In addition, our findings indicate that the expression levels of genes associated with tegument maintenance are the most pronounced in female_single worms.

The herein observed tuft-like repulsion of the opsonized surface of female worms was described so far during the early development of schistosomula. As part of the transformation process during skin penetration, the outer cercariae membrane is transformed into microvilli that are subsequently shed. Meanwhile, multilaminate vacuoles are translocated from the subtegumental cell body into the tegument to build up a new outer membrane of the tegument (Jones et al. 2004; Ressurreição et al. 2016; Hockley and McLaren 1973). It is conceivable that the observed repulsion of the tegument of female worms is part of a rebuilding process from the inside out and old components are repelled to the outside (Wilson and Barnes 1977). Cercariae and newly transformed schistosomula are highly sensitive to the binding of complement factors and subsequent cytotoxic mechanisms. However, this vulnerability is lost within a few hours (Marikovsky et al. 1990). It has also been described that adult schistosomes, transferred from one host to another (here from mouse to human), survive with adaptation difficulties at first (Smithers et al. 1969). With regard to adult female schistosomes, it is most likely that the tegument-repulsion after contact with NHS is a kind of adaptation to the host/hostile environment.

Single sex infection studies revealed that male schistosomes undergo normal morphological development, while female worms show stunted and undeveloped maturation (Popiel and Basch 1986; Loverde and Chen 1991; Kunz 2001; LoVerde 2002). To our knowledge, this is the first time that the ultrastructure of the tegument of adult worms out of a pair has been described in direct comparison to worms originated from single-sex infections following confrontation with NHS of a new host. In our study, female and male schistosomes out of a pair showed marked morphological changes due to the incubation with NHS compared to NHSi. We observed a great surface enlargement seen by the increasement of numbers of pits per area tegument combined with an altered structure of the pits below the surface. This invaginations of the tegumental surface might be a part of caveolae-like structures known to play a role in endocytosis and transcytosis of plasma proteins (Racoosin et al. 1999; Cheng and Nichols 2016). It is known that adult schistosomes utilizes components from the host’s blood for their own purposes, such as proteins and carbohydrates (Brindley et al. 1997; Camacho and Agnew 1995; Halton 1997). The observed surface enlargement following the NHS incubation described by us could thus indicate increased metabolic activity of adult schistosomes. Caveolae were also been described to be part of specific signaling pathways or mechanosensitivity and therefore potent communication tools at the interface between the adult schistosomes and their host (Parton and Simons 2007).

The enlargement of the tegumental surface is most pronounced in female schistosomes in response to NHS after PZQ and goes in line with the ability of female schistosomes to recover faster than male worms after in vitro PZQ treatment. It has been shown that female_single are largely and male_single moderately resistant to treatment with PZQ when exposed to the drug and an incubation in drug-free medium afterwards (Pica-Mattoccia and Cioli 2004; Inal 2004). Our data confirmed these findings. It was also demonstrated that male cercariae of *S. mansoni* had significantly higher tail-shedding rates than female cercariae when exposed to the same concentration of PZQ (Liang et al. 2010). The herein demonstrated motility scores showed a faster restoration of the motility for female schistosomes compared to males. Park et al. (2019) demonstrated that PZQ activates a schistosome transient receptor potential channel, Sm.TRPMPZQ, present in schistosomes and other PZQ-sensitive parasites. However, nothing is known so far about a gender-specific protein expression of this channel following a PZQ challenge. In our study, NHS seems to promote regeneration after PZQ in contrast to NHSi. This effect is most pronounced in female schistosomes. It seems that females, unlike males, can utilize a component from active serum to recover after PZQ challenge. However, so far, we can only speculate on possible mechanisms. Male and female_single schistosomes in NHSi after PZQ showed pronounced alterations of the tegument and the structures below including muscle shrinkage, vacuolization, blebs, and production of membrane bodies after PZQ exposure. Due to the sublethal dose of PZQ, used in this study, these alterations are less severe than described previously (Shaw and Erasmus 1983; Mendonça et al. 2016; Matos-Rocha et al. 2016). Taken into account the increased membrane activity in combination with the pronounced surface enlargement of female worms, it could be assumed that this might be advantageous for the survival within the human blood as well as the regeneration capacity after PZQ compared to male worms.

In contrast to our morphological observations on changes of the tegument of female and male worms after incubation in NHS, on molecular level only female worms from unisexual infection showed significant changes of genes associated with tegument maintenance. For example, a zinc finger protein called ZFP-1-1, with high expression levels in female_single after 0.5 h in NHS, has been proven to be crucial for the control of tegumental neoblast-driven maintenance and tegumental cell specification (Wendt et al. 2018). Comparable expression levels were presented for genes encoding for vesicular integral-membrane protein 36 (Vip36), shown to participate in the complex secretory activity of tegument proteins (Ornelas et al. 2017), family S28 unassigned peptidase (S28), tetraspanin-2 (TSP-2), calpain, and dysferlin. The latter have been identified as part of protein complexes known as tetraspanin-enriched microdomains that mediate a range of processes at the surface of the plasma membrane (Jia et al. 2014; Schulte et al. 2013). In addition, calpain was shown to modify the worm’s local environment by cleaving the host clotting protein fibronectin and the coagulation protein high molecular weight kininogen to ensure parasite survival, and dysferlin is reported to be involved in membrane repair mechanisms (Wang et al. 2018; Xiong et al. 2013). In contrast, the expression of *enolase* was only increased in male_single after 0.5 h in NHS. The tegumental enzyme enolase is able to promote blood clot degradation around the worm (Figueiredo et al. 2015). Further, it has been shown that host proteins are able to bind to enolase and are integrated into the outer membrane of the tegument suggesting to be an important part of their immune evasion strategy (Angeles et al. 2020). In principal, we herein observed higher gene expression levels for all genes tested after contact with NHS compared to NHSi. This might be at least related to adaptation processes following host change (mouse to human serum). The tuft-like repulsion of the female tegument, the faster regeneration toward PZQ, and the upregulation of genes associated with tegument maintenance after contact to NHS might indicate that female worms are equipped with different protection mechanisms against soluble factors of NHS. Due to the observed faster regeneration in NHS after PZQ compared to NHSi, one might speculate on the ability of female worms to utilize serum proteins for their survival in contrast to male worms. Nevertheless, more in-depth and comprehensive analyses must be carried out in order to understand how the tegument of both worm sexes is interacting with and is influenced by the host serum.

## Conclusion

We herein demonstrated that following the incubation in human serum, female schistosomes repel their outer opsonized surface. We demonstrated that males and females out of a pair display pronounced morphological changes at their surfaces following incubation with NHS seen by an increased number of pits per area tegument and below resulting in an altered structure of the pits. Furthermore, we could show that female worms are significantly more resistant to incubation with PZQ than male worms. The female tegument showed a pronounced enlargement of their surface structure compared to male worms with a great regenerative capacity after incubation in NHS and PZQ. Following PZQ incubation, male worms display a degeneration of the tegumental and subtegumental layers as well as muscle shrinkage with widespread vacuolization leading to a prolonged recovery time compared to the female worms. We were also able to show that both female and male schistosomes regenerate poorly or not at all after PZQ in NHSi. On molecular level, analyses of expression profiles of tegument-associated genes showed an upregulation of genes involved in the maintenance of the tegument that was most conspicuous in female_single worms after NHS. Our findings provide evidence that female schistosomes evolved different evasion strategies toward the host’s immune system in comparison to male schistosomes that might lead to more robustness and has to be taken into account for the development of new anti-schistosomal drugs.

## Supplementary information

ESM 1 (DOCX 19.3 kb)
