# Supplementary material for: Human serum activates the tegument of female schistosomes and supports recovery from Praziquantel
Source: Parasitol Res. 2020 Dec 1;120(1):209–21. doi: 10.1007/s00436-020-06968-x (PMC7846515; doi:10.1007/s00436-020-06968-x)
Supplement: Supplementary file 1 — (DOCX 19.3 kb) [file 436_2020_6968_MOESM1_ESM.docx]

**Supplementary Material**

**Table 1** Primers and probes used for the detection of gene expression levels of tegument-specific proteins.

| **Accession No.** | XM_018797513.1 | M74233.1 | XM_018791830.1 | XM_018791741.1 | XM_018793159.1 | U30175.1 | XM_018789969.1 | AF521091.1 | XM_018793276.1 | XM_018794729.1 |
| --- | --- | --- | --- | --- | --- | --- | --- | --- | --- | --- |
| **Probe** | 5’-FAM-TGAGGCTCGT GAAGATCTGG-TAM-3’ | 5´-FAM-TGAGCTTCAC TGCAGATGGA-TAM-3´ | 5′-FAM-TCATGGTGTAT TAGCTGTTGGT-TAM-3′ | 5´-FAM-GGAAAGTTGC TGAACCTGCA-TAM-3´ | 5′-FAM-AAAAACGGAG AAACTGGGCG-TAM-3′ | 5’-FAM-TCGTCTCCA ACAATTTGAA-TAM-3’ | 5′-FAM-ACCAAACGGA CCCATCTTCT-TAM-3′ | 5´-FAM-CTGGAGCGA AATTGAACCCC-TAM-3´ | 5´-FAM-GGCCAGCAT TCGGTAGTAGA-TAM-3´ | 5´-FAM-TTGTCATTGC TTCCTGCCAC-TAM-3´ |
| **Reverse Primer** | 5’-ACTCTTCACC TTCCCCTTCG-3’ | 5’-CGTTACGTTG CCATTGACCA-3’ | 5′-ACCCCATGTA GTACCCCAAC-3′ | 5’-AAACCGTCTT CATCGCATGG-3’ | 5′-GCACTCAGGC GAACTTTCTC-3′ | 5’-CGCTTTGGATT GGTAACAGTCAA-3’ | 5′-TCAGCAAACA GGACGAAAGC-3′ | 5’-ATGCATGCTC CAAACATCCC-3’ | 5´-AAGAAAACG GCTAACCCACG-3´ | 5´-GCATTTGTCG ACATACCCGT-3´ |
| **Forward Primer** | 5’-TGGGCGCGT CTAGATCATAA-3’ | 5’-GTGCATGGTG TGATGGTTCA-3’ | 5′-ACTATGGACCA ATTTCAGTTGCT-3′ | 5’-TTCGTACCCC AACAAGTCCA-3’ | 5′-CGCAGATTGT TGAAGAGGCA-3′ | 5’-GGCCCAAACTAA CTTCTTCAACAAA-3’ | 5′-GTGGCCACCG AAAGAAAACT-3′ | 5’-TGGCTCTTGG TTGTGGGTAT-3’ | 5´-AGCCTCAGTT TGTACGCCTA-3´ | 5´-CAGCAACAAC AACAGTCCCA-3´ |
| **Gene** | *sat1* | *Smp_157500* | *Smp_139160* | *Smp_141010* | *Smp_003230* | *eno* | *Smp_071610* | *Smp_024820.2* | Smp_174580 | Smp_049580 |
| **Protein** | α-Tubulin | Calpain | SmCL2-like peptidase | Dysferlin | Endophilin B1 | Enolase | Family S28 unassigned peptidase (S28 family) | Tetraspanin-2 | Vesicular integral-membrane protein vip36-related | Zinc finger protein-1-1 |
